# Improving transgender health by building safe clinical environments that promote existing resilience: Results from a qualitative analysis of providers

**DOI:** 10.1186/s12887-015-0505-6

**Published:** 2015-11-18

**Authors:** Carlos G. Torres, Megan Renfrew, Karey Kenst, Aswita Tan-McGrory, Joseph R. Betancourt, Lenny López

**Affiliations:** Harvard Medical School, Boston, Massachusetts USA; Partners HealthCare, Boston, Massachusetts USA; Massachusetts General Hospital, Boston, Massachusetts USA; Disparities Solutions Center, Massachusetts General Hospital, Boston, Massachusetts USA; Mongan Institute for Health Policy, Massachusetts General Hospital, 50 Staniford St, Suite 901, Boston, Massachusetts 02114 USA

**Keywords:** Transgender health, LGBT health, LGBT youth, Transgender youth, Health disparities, Resilience

## Abstract

**Background:**

Transgender (TG) individuals experience discordance between their sex at birth and their gender identity. To better understand the health care needs and characteristics of TG youth that contribute to resilience, we conducted a qualitative study with clinical and non-clinical providers.

**Methods:**

In-depth interviews were conducted of providers (*n* = 11) of TG youth (ages 13–21). Convenience and purposive sampling were used to recruit participants in the Boston area. All interviews were audio-recorded and transcribed verbatim. An interview guide of 14 open-ended questions was used to guide the discussion. A grounded theory approach was utilized to code and analyze the data, including double-coding to address issues of inter-rater reliability.

**Results:**

Five primary themes emerged: 1) resilience of TG youth 2) lack of access to services that influence health, 3) the critical role of social support, 4) challenges in navigating the health care system, and 5) the need for trans-affirming competency training for providers and frontline staff.

**Conclusion:**

The findings of this study show that providers recognize multiple barriers and challenges in the care of TG youth. However, they also identify the resilience exhibited by many youth. We propose that providers can further enhance the resilience of TG youth and help them flourish by offering them necessary resources via the creation of safe and welcoming clinical environments.

## Background

Transgender (TG) individuals experience discordance between their sex assigned at birth and their gender identity. The word “transgender” has become an umbrella term encompassing all people whose gender identity or expression does not conform to that typically associated with the sex they were assigned at birth [1]. Prior studies estimating the prevalence of TG youth in the general population have varied from 1 to 3.5 % [2, 3].

Research about TG youth remains limited [4, 5]. Existing studies have focused primarily on TG adults. Although there may be similarities among adults and adolescents, TG youth represent a population with unique needs that have not been fully explored. Prior studies have shown that TG youth often face discrimination and victimization since they do not adhere to conventional gender expectations [5, 6]. In a study of experiences of TG youth accessing and utilizing health services, Grossman & D’Augelli (2006) noted four key barriers to health: 1) lack of safe environments, 2) poor access to physical health services, 3) inadequate resources to address mental health concerns, and 4) lack of support from their families and communities [7]. Similar to LGB youth, TG adolescents are at increased risk for low self-esteem, depression, suicide, family and peer rejection, physical abuse and trauma, and inadequate housing [2, 6]. Therefore, TG youth are considered a vulnerable and medically underserved population.

Despite significant challenges, there is evidence that TG adolescents also possess positive attributes for overcoming existing barriers. Resilience is broadly defined as the ability to “bounce back” and successfully manage difficult experiences [8]. It has gained considerable attention since researchers observed that children and youth could cope, adapt, and succeed in spite of adversity [9]. Resilience theory is based on strengths rather than deficits and focuses on promotive factors (assets or resources) that enable adolescents to overcome the negative effects of risk exposure. *Assets* are the positive factors that reside within an individual (i.e., competence, coping skills, self-efficacy) whereas *resources* are the external positive factors (i.e., parental support, adult mentoring, community organizations) that help youth overcome risk [10]. Similarly, research on the elements of positive human development has identified a set of interrelated experiences, relationships, skills, and values that are associated with reduced high-risk behaviors and increased thriving behaviors [11]. Furthermore, studies have shown that adolescents who find a community of others who share their interests may do better psychosocially than those who are isolated [12]. One study on TG youth identified key *assets* correlated with their resilience, including personal mastery, self-esteem, and perceived social support [13]. Providers were not mentioned as contributors to resilience for these TG youth.

There is a paucity of literature from the perspective of providers who care for TG youth [14, 15]. In order to better characterize the complex interplay between personal attributes that promote resilience and environmental barriers, we conducted a qualitative study of TG youth providers. Providers may offer unique insights into the lives of TG youth and how to best structure resources for this population.

## Methods

We conducted a qualitative study to better understand provider’s perspectives on the health care needs and barriers to care for TG youth in Boston. The study was approved by the Harvard Medical School Institutional Review Board. We obtained written consent from participants prior to the interviews.

We used convenience and purposive sampling to recruit participants known in the Boston community for their professional work with TG youth. Providers included clinical staff (two psychiatrists, two behavioral health clinicians, one nurse), researchers (one epidemiologist, one advocacy expert), and trained community educators. Five participants (45 %) self-identified as TG. *Youth* were defined as individuals between the ages of 13 and 21 (per Institute of Medicine 2011). Based on an exhaustive literature search, we developed an interview guide consisting of 14 open-ended questions (Appendix 1). In 2011, we conducted individual interviews with clinical and non-clinical providers until thematic saturation was reached (*n* = 11). All interviews were audio-recorded, transcribed verbatim with personal identifiers removed, and lasted approximately 1 h. Providers were asked to sign an informed consent if they wished to participate; they were informed that they could withdraw consent at any time. The interviewer had training in qualitative interviewing techniques.

We used Strauss and Corbin’s grounded theory approach for the coding and analysis [16]. Three of the co-authors reviewed five transcripts to identify the main themes. Using inductive coding, we identified 13 themes that were then reduced to eight after testing the draft scheme on three transcripts. Once the major concepts were established, we completed detailed coding to identify sub-themes, connections between codes, and inclusion and exclusion criteria (Appendix 2). We double-coded three transcripts to ensure coding agreement and address inter-rater reliability. Using multiple coders is a standard and dynamic process commonly used in qualitative research to cross-check coding strategies and interpretation of data by the research team [17]. There was 80 % agreement among the coders. Differences in coding were resolved with team discussions, and we used Atlas.ti software [18].

## Results

Interviews of providers who interact closely with TG youth highlight their unique health care needs and distinctive characteristics. Five primary themes emerged: 1) resilience of TG youth 2) lack of access to services that influence health, 3) the critical role of social support, 4) challenges in navigating the health care system, and 5) the need for trans-affirming competency training for providers and frontline staff. While the most frequently addressed themes included the lack of access to general services and the importance of social support, the most salient theme included the resilience displayed by TG youth.

### Theme 1: Resilience of TG youth

A prominent theme was the resilience of many TG youth despite the obstacles faced in their day-to-day lives. This resilience was attributed to the degree of social support, role models/mentors, and family acceptance, as well as the goals and aspirations of TG youth. One participant stated: “What is different about them? They are resilient. They have a goal. They believe that there is something worth fighting for.”

Participants also noted that resilient TG youth tended to have role models and mentors who were also TG that they could turn to for questions, advice, and support. One mental health provider fostered these connections by organizing a panel for TG youth and their families to hear from TG adults who had overcome adversity: “One of the panelists was a trans male in his early thirties who was married and had a child with his partner … I wanted people to see that there are positive outcomes for their children rather than what they see in shows or what they hear in the news or statistics.” This provider aimed to enhance the resilience of TG youth by creating opportunities for TG youth to meet and identify with successful role models.

### Theme 2: Lack of access to services that influence health

#### Mental health

When we asked providers about the health care needs of TG youth, they reported that access to mental health services was especially challenging. TG youth often experience mental distress and suicidal behavior due to a lack of acceptance. One participant stated, “TG youth are often grappling with how to live in a world with a set of parents that don’t want them to be who they are. They also live in a society that is stigmatizing and discriminatory. This ought to put these kids at increased risk for mental health issues.” Participants reported that TG youth suffer from depression, PTSD, anxiety, and substance abuse at higher rates compared to other adolescents.

#### Safety, housing, and employment

Participants emphasized the importance of housing, violence, and employment in the lives of TG youth. For instance, one provider stated: “We cannot talk about the health of these youth and not talk about discrimination, violence, and housing…We need to focus on all of those things because I’ve seen it all affect their health.” One participant explained why housing was so important: “I put housing as a health care need because you need housing to have adequate health. You need a place to sleep and feel rested and healthy, to shower and feel safe.” Lack of employmen was another barrier to accessing care highlighted by providers. One participant explained, “Unemployment is one of the biggest barriers for care… if your legal documents don’t match with how people are seeing you, people don’t get hired.” Consequently, providers reported that TG youth must feel safe at home and in their communities in order to be proactive about their health.

#### Medical transition

Many providers mentioned that TG adolescents are overall physically healthy and that their main concern was “proving their gender.” In order to “pass” as the desired gender, TG youth may undergo medical interventions that consist of cross-gender hormone therapy and/or surgeries. By “passing,” many TG youth gain a sense of belonging and confidence. However, some TG youth may not wish to “transition.” One participant stated: “There are so many ways to be TG. Some people don’t want technologies. TG youth these days are so diverse, and they express themselves in different ways.”

#### Health insurance

Inadequate access to care is further complicated by insurance issues; most insurers do not cover TG care. Many insurance companies have clauses that do not cover TG related services such as hormones (including GnRH agonists which can be extremely costly) and sex-affirming surgery. Providers described that their patients often have to work multiple jobs (including sex work) to save enough money to pay for treatments out of pocket. They expressed disappointment with insurance companies who often do not understand how serious the need for medical services may be for some TG youth. Some providers manage to find ways to overcome insurance barriers such as providing hormones at wholesale cost or changing the gender on the patient’s paperwork so they qualify for hormones under a different medical reason.

### Theme 3: The critical role of social support

Another reoccurring theme was the crucial role of social support, including family, school, and the broader community in providing acceptance, protection from violence, and housing. One provider illustrates this by stating: “We call it the TRIFECTA: you need to have the parent, the community, and the school. You need to have all those three things to create a sense of safety and empowerment. If you only have one and not the other two, everything goes awry.”

Acceptance by their families is a key element in the well-being of TG youth. Many youth are rejected by their families after coming out as TG. The lack of acceptance often leads to homelessness, which negatively affects TG youth, who “are often cast out from their families with no place to live, so they turn to the streets,” stated one provider. “Sometimes you have to make the choice: do I stay with my family or do I live my life? Sometimes you have to choose yourself and not have a family, which is heart-breaking.”

Another crucial component is parental consent, which is required for TG youth under 18 who are interested in medically transitioning. “In order to get the treatment you need, you need parents behind you…we need to teach [parents] that by not allowing their children to be who they are, they are hurting them; that is bad parenting.” A therapist who works closely with parents stated that giving consent is a daunting process for parents for a variety of reasons: “Each set of parents has their own struggles: for those with kids under 18, they feel that they must keep their kids safe…they are afraid that they will consent and their children will change their minds…They must initial every possible risk factor that could happen, which is scary for them.”

Providers also noted the high rates of victimization and bullying that TG youth must endure in schools. A mental health provider described leading a therapy group for TG youth where “all of the kids in the group expressed being bullied at school for being trans. Their peers were just awful, and these were just little kids.” These experiences negatively affect TG youth and prevent them from focusing on their education. Participants identified finishing school as a very important indicator of future success and job attainment, so they emphasized the importance of providing TG adolescents with support in completing their education.

Participants also highlighted the importance of role models in the community for the development of TG youth. The community was described as vital in providing connections and networking. Providers often commented on the importance of support groups: “It was an opportunity for folks to get together and talk about transitioning, family issues, problems getting healthcare, etc. People could share ideas and connect. ‘I know that this doctor is friendly.’ There is not a list of it or anything. People access doctors through these networks.”

### Theme 4: Navigating the complex medical system

Navigating the health care system is a major barrier for TG youth under 18 who often depend on their parents for health insurance, transportation, and consent. An important component of this challenge is the lack of agency via-a-vis the healthcare system at a young age. One provider powerfully describes this: “Understanding how to navigate health care systems as a child is almost impossible. I mean no one sits you down and teaches you how to do that…You acquire that knowledge as you grow older and based on the environment that you grew up in and based on your experiences.” Once they take the leap to seek care, TG youth face a complex medical system that requires many interactions (with secretaries, insurance personnel, pharmacists, specialists, etc.). All of these points can be avenues of great help or cause great stress. Another participants states: “When we think of going to the doctor, there are all those barriers to getting to the doctor: making the appointment, walking to the doctor, physically getting inside the door…How do they handle having a male presentation but having female on the paperwork and you showing up for a pap smear? Each point is a potential barrier to care.”

### Theme 5: Education and training of all staff

Even when TG adolescents have parental support and resources, providers are often not trained or willing to provide care to TG youth due to stigma, lack of education, or the scarcity of evidence on the long-term effects of hormones or side effects of other treatments. One participant stated, “There are very few providers who besides being comfortable working with trans youth, with a stressed emphasis on the youth part, that are actually good at it, well informed, keep up with research and do the best that they can for their patients just like they would do for anyone else.” Participants hypothesized that the lack of providers who work with TG youth may result from a fear of being sued if patients regret their decision to transition or fear of causing harm.

Participants emphasized the need for trans-affirming competency training for providers and frontline staff about providing high-quality care for TG patients. They noted that patients and their families must often educate their providers about what it means to be TG, which places undue burden on the patient. Participants expressed that educating providers is an important task that should not be the responsibility of TG patients. “I don’t want TG people to educate their doctors every time…it can be exhausting.”

Additionally, safe clinical care environments in which patients are addressed by their desired name and pronouns is key to ensuring culturally competent care. Intake and clinical forms must be inclusive of all genders. According to participants, creating a welcoming environment includes offering gender-inclusive bathrooms and information about resources available to TG youth (i.e., pamphlets, brochures, etc.). Participants further expressed that a focus on creating safe clinical environments would facilitate TG access to medical services.

The inpatient setting can be a challenge for both TG youth patients and providers. Some participants recommended decreasing the number of interactions that TG patients have. Another suggestion consisted of posting an alert in the patient’s chart that states they are TG, their preferred name and pronoun, and any other information that may be useful (similar to an allergy alert). Another provider recommended the health navigation model, “where you walk in and you have an advocate that can guide the person throughout the entire encounter. I say this even informally to friends…bring a friend or someone to help you navigate these points of contact…”

## Discussion

This study provides several important and novel findings. Instead of focusing solely on risk factors encountered by TG youth, our study highlights the central role of resilience and the importance of creating nurturing environments to help enhance this resilience. Resilience is a dynamic concept that focuses on strengths (*assets* or *resources*) that enable adolescents to overcome the negative effects of risk exposure [10]. Although it may be impossible to influence or change a person’s internal psychological assets, it is possible to enhance protective resources [19]. Research on resilience and youth shows that protective resources buffer the impact of risk factors on the child by building communities that support human development based upon caring relationships and meeting youth’s needs for belonging and stability [19, 20]. Studies have shown that this can happen in different ways and involve multiple people. For instance, parents or mentors can provide general life guidance while providers can promote positive behavior, identify risks, and implement programs that involve the family and mobilize community resources [9, 21]. Therefore, we propose that creating nurturing environments by providing much-needed resources may help enhance this resiliency specifically among TG youth. Providers should approach TG youth from a strengths perspective, identifying internal assets and providing external resources that can help youth flourish.

**Fig. 1:**
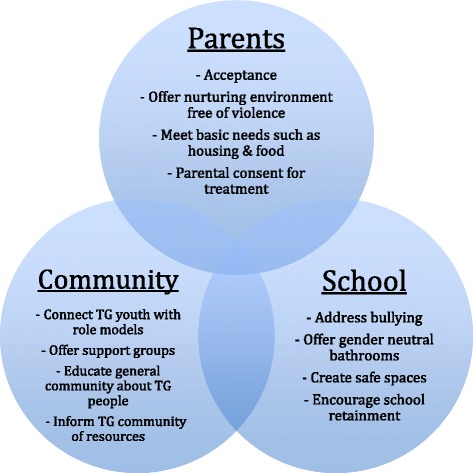
The Crucial Role of Parents, Community, and School in Creating Safe Environments for Transgender (TG) Youth; This figure presents three separate but overlapping spheres of influence in a youth’s development, including parents, school, and community. Positive features or suggestions are listed within each component that would help create an all-around safe environment for TG youth

Our study supports the critical role of social support from family, school, and the community (Fig. 1). Their roles include providing acceptance, safety from violence, and housing. Another crucial component is parental consent, which is necessary for TG youth under 18 who are interested in medical interventions. Our participants highlighted the importance of having role models and mentors in the community for the development of TG youth. A provider who self-identified as TG stressed the importance of support groups for TG youth: “You don’t have any rights in this country. People can fire you for being TG. You do not have to be housed. You are pathologized…So, it’s nice to have other people around that are like you and feel the same way and have similar experiences.” Similar to some researchers and members of the TG community [22], our participants challenged the premise that TG identity is a psychiatric pathology. Although the term “gender identity disorder” has changed to “gender dysphoria” in the DSM V to remove the connotation that the patient is “disordered,” it is arguable whether the term should even be used [23]. Regardless, providers should encourage participation in group meetings and organize parent skill trainings and support groups to encourage parents to provide supportive environments for their children [24]. Such programs provide a supportive community for parents, where they are encouraged to unconditionally value their child, acknowledge their differences, assist the child in navigating schools and society, and advocate for changes in the family and community [25–27]. Additionally, Central Toronto Youth Services, an organization that advocates on behalf of TG adolescents, offers a resource guide for parents of TG youth entitled *Families in TRANSition* [28]. These services have the potential to increase resilience by offering information and fostering the necessary social support for TG youth. A key challenge involves overcoming potential barriers to parental participation.

Participants in our study reported the unique challenge of navigating the health care system for TG youth under 18, who depend on their parents for health care services. One provider stated: “For people under 18, the biggest challenge is getting to the right types of health care organizations because they rely on parents for health insurance and transportation and permission to initiate hormone use or services that would facilitate a physical transition.” An important component of this challenge is the lack of agency that comes with being young. One may consider adopting the patient navigation model to guide TG youth through our complex medical system.

Participants also identified basic health care needs of TG youth including mental health, preventative services, social services (such as housing), endocrinological care, and transitioning. Our findings are consistent with prior research that has identified a general lack of access to health services and a lack of continuity of caregiving by families and communities [7].

Accessing or navigating the health care system may be especially challenging for TG youth with multiple identities. One participant stated: “when you put a different combination of oppressions (gender identity, race, undocumented), it’s like a boiling pot of awfulness. And I think we health care providers should be aware of this. We need to find them providers who are savvy about the trans stuff, but also be competent around issues of social class, ethnicity, disability status etc.”

Trans-affirming competency training for all providers may also promote resilience. One of the strengths of this study is the diversity of service providers who were interviewed. This also shows how trans-affirming trainings do not only apply to medical providers [29], but also to the many disciplines involved in the care of TG youth, which include social work [30], hospital staff [31], and nursing [12]. It is well documented in the literature that strong patient-provider relationships are of vast importance. For instance, one study showed that individuals with HIV who had a supportive attachment figure with their providers (including nurses, physicians, case managers, etc.) had positive outcomes related to disease management and overall well being, demonstrating the power of providers in contributing to the development of resilience [32]. It may be reasonable to hypothesize that this powerful relationship is also important to other disenfranchised groups such as that of TG youth patients and their providers.

Therefore, our study reveals the need for more providers who offer TG specific services to youth in a safe clinical care environment in which patients are addressed by their desired name/pronoun and intake and clinical forms are inclusive to all genders. “The medical environment should be welcoming and friendly: what is on the walls, who is the receptionist, how do they treat you when you walk in.” There are some organizations that provide resources and trainings for providers such as Gender Spectrum which features tips for instituting gender inclusive practices, best practices for frontline staff, and gender affirmative signage [33, 34].

Prior studies of LGBT youth have shown that they wish to receive private and confidential services, to be treated with respect and honesty, and to be seen by providers who are well trained and have good listening and communication skills [35]. Our participants also advocated for the establishment of “Centers of Excellence for TG Health” that specialize in providing health care for this group (primary care services, case management, counseling, support groups, etc.). Such comprehensive programs have started to exist in large metropolitan areas. One specific example is “The Child and Adolescent Gender Center” in San Francisco, which provides TG youth patients and their families access to mental health, endocrine medical care, parent support groups, case management, and legal and educational advocacy services [36]. Their endocrine care is based on guidelines compiled by the World Professional Association for Transgender Health, which include the standard of care for diagnostic assessment, psychotherapy, real-life experience, hormone therapy, and surgical therapy [14, 37]. One of the first studies that provides demographic and clinical data on TG adolescents treated at a pediatric center in the United States with pubertal suppressive therapy and/or cross-sex hormone therapy shows significant improvement in psychological functioning following the medical intervention [38]. Because locating specialized services for TG youth can be challenging, Hsieh and Leininger (2014) have compiled a list of clinics organized by geographical area (covering US and Canada) [39].

Nevertheless, our study has several limitations. Due to the small number of participants interviewed, we cannot assume that these findings are representative of all TG providers. Given the limited number of providers who care for TG youth, we used convenience and purposive sampling which further limits generalizability and presents issues around selection bias. Since most of our participants were strongly trans-affirming, their non-judgmental nature and knowledge shared probably differs from that of other TG youth providers. Moreover, the data gathered is from a single city that does have some services for TG youth in place and may not reflect additional issues of importance to TG youth in areas with fewer services. However, it is important to note that the participant heterogeneity provides multiple perspectives and thus a more holistic view of the barriers and facilitators to resilience in this population.

**Fig. 2:**
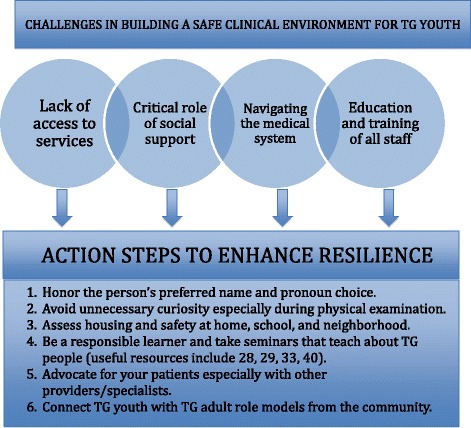
Recommendations for Improving Transgender (TG) Care Based on Participant Interviews; This figure outlines the challenges and barriers associated with building a safe clinical environment for TG youth and proposes six action steps that can be taken to enhance resilience within this population

This study addresses the need for research among professionals who work on the frontlines of TG health and related social services [2, 40]. We offer new insights regarding the needs and barriers to care for TG youth by providing the diverse perspectives of clinical and non-clinical staff. Their knowledge provides insight into the experiences of TG youth that often are not captured in research surveys. We provide a complementary view to the limited data available on TG youth. Importantly, our participants suggested nine key recommendations for clinical practice to promote resilience (Fig. 2).

Instead of focusing on the risk factors encountered by TG youth, we propose focusing on the development of external resources of TG youth via parenting skills, adult mentorship, fulfilling TG and non-TG health needs, and helping them navigate complex health care systems. Future efforts need to focus on creating a holistic and supportive clinical environment that promotes resilience among TG youth.

## Conclusion

The findings of this study show that providers recognize multiple barriers and challenges in the care of TG youth. However, they also identify the resilience exhibited by many youth. We propose that providers can further enhance the existing resilience of TG youth by focusing on the development of external resources of TG youth. In this manner, they can contribute to their positive development and help them flourish.

## Appendix 1

### Interview guide

1. Can you describe what you do?
2. What role do you (and your organization) play in providing care to the TG youth community?
3. How did you become involved in doing this?
4. Can you describe the patients who seek care with you? (ie. how are they economically, ethnically, socially?)
5. Do you work/interact with TG youth patients? (Defined as patients ages 13–21) *Please focus your answers to what you see in this group specifically*.
6. Is there a particular story that comes to mind or that has impacted your work?
7. What do you consider to be the most important health care needs (if any) of TG youth?
8. Do you think these needs are different compared to those of TG adults?
9. What characteristics would you attribute to a physician/caretaker who provides excellent quality of care to this population?
10. How would you describe the quality of health care received by TG youth?
11. What do you think are the main barriers (if any) to seeking medical care for the TG youth?
12. Can you describe some of the barriers you have encountered (if any) in providing the best quality of care to TG youth patients? *Please offer specific examples if possible*.
13. What do you think can be done to make the health care services or resources more TG-friendly?
14. Are there particular health services that are more difficult to access by the TG youth community?

## Appendix 2

**Table 1:** Coding Scheme: Main themes, sub-themes and inclusion/exclusion criteria

Code title	Code	Inclusion criteria	Exclusion criteria
1. Health Care\Barriers/Needs			
*Non-TG specific health barriers/needs*	1.A	a) Mental Health	
		b) Preventative Health Services	
*TG-specific health barriers/needs*	1.B	a) Medical transition	Gender identity disorder as a pathology under 2.C
		b) Hormone therapy	
		c) Surgery	
		d) Standards of care	
		e) Gender identity diagnosis	
2. Other Barriers			
*Socio-Demographic*	2.A	a) Housing	
		b) Geographic location	
		c) Transportation	
		d) Financial	
*Clinical Encounter Barriers for TG*	2.B	a) Access to care	References to navigating health care system coded below under 6.A
		b) Health insurance	
		c) TG-doctor-patient encounters	
		d) Lack of available information	
*Other Barriers*	2.C	a) Violence/abuse/safety	Other issues related to safety of school environment coded under #3
		b) Discrimination	
		c) Stigma	
		d) Employment	
		e) Government issued identification	
		f) Legal/arrest	
		g) Bathroom access	
		h) Education	
		i) Substance abuse	
3. Social and Family Support			
Family	3.A	a) Family composition/structure	
		b) Parental support	
		c) Lack of family support	
		d) Parental Consent	
Print and Social Media	3.B	a) Films, TV specials, internet, etc.	
School	3.C	a) References to school environment	Education as barrier coded above in 2.C
4. Community-Based Institutions and Resources			
Community-based health and social support services	4.A	a) Community-based events, services, support groups	
		b) References to specific organizations	
5. Recommendations: Characteristics of Excellent Care			
Provider Education (staff specific)	5.A	a) Cultural competency training and TG awareness and education for all staff	Direct references to cultural competency and providers
Clinical Environment	5.B	a) Welcoming environment	
		b) References to supportive environment	
		c) List of resources available for patients	
Approaches to TG Care	5.C	a) Psycho-social approaches to care that may include risk reduction models, peer-to-peer, mobile testing, holistic approach/comprehensive care, involving TGs in the delivery of services	
6. Health Care System			
Difficulties Navigating Health Care System	6.A	a) References to challenges navigating the system	
7. Personal Experiences			
Role of Personal Experiences Shaping TG Advocacy/Research	7.A	a) Includes references to TG self-identification	
8. Other Issues			
Research/lack of on TG	8.A	References to need or lack of research on TG	
Complex Identities & Diversity of TG	8.B	Includes references to multiple identities	
Resilience of TG Youth	8.C	References to TG youth being resilient in face of challenges	
Advocacy	8.D	Specific references to advocacy, leadership development, legislation/policy	Community-based social services are coded under 4.A
Characteristics of Clients Served	8.E	Description of clients served at participant’s institution	Diversity of TG in general is coded under 8.B
Differences in Care Needs between Adults and Youth	8.F	Description of ways in which care needs or care services differ for youth vs. adults	
Great Example	8.G	Examples, stories about TG youth that would be ideal to highlight in paper	
Other	8.H	May include: a) Mistrust of doctors by TG Community b) Jail/Prison	

## Abbreviations

TG: Transgender (as defined in manuscript)
